# Changes in cell differentiation and proliferation lead to production of abzymes in EAE mice treated with DNA–Histone complexes

**DOI:** 10.1111/jcmm.13850

**Published:** 2018-09-28

**Authors:** Kseniya S. Aulova, Ludmila B. Toporkova, Julia A. Lopatnikova, Alina A. Alshevskaya, Sergey E. Sedykh, Valentina N. Buneva, Thomas Budde, Sven G. Meuth, Nelly A. Popova, Irina A. Orlovskaya, Georgy A. Nevinsky

**Affiliations:** ^1^ Institute of Chemical Biology and Fundamental Medicine Siberian Branch of the Russian Academy of Sciences Novosibirsk Russia; ^2^ Institute of Fundamental and Clinical Immunology Siberian Branch of the Russian Academy of Sciences Novosibirsk Russia; ^3^ Institut für Physiologie I Westfälische Wilhelms‐Universität Münster Germany; ^4^ Department of Neurology Westfälische Wilhelms‐Universität Münster Germany; ^5^ Institute Cytology and Genetics Siberian Branch of the Russian Academy of Sciences Novosibirsk Russia; ^6^ Novosibirsk State University Novosibirsk Russia

**Keywords:** C57BL/6 mice, catalytic antibodies, colony formation, cytokines, eae model, hematopoietic progenitors differentiation, immunization with DNA–histone complex

## Abstract

Experimental autoimmune encephalomyelitis (EAE)‐prone C57BL/6 mice are used as a model of human multiple sclerosis. We immunize mice with myelin oligodendrocyte glycoprotein (MOG), DNA–histone and DNA‐methylated bovine serum albumin (met‐BSA) complexes to reveal different characteristics of EAE development including bone marrow lymphocyte proliferation and differentiation profiles of hematopoietic stem cells. Immunization of C57BL/6 mice with MOG_35‐55_ results in the acceleration of EAE development. Anti‐DNA antibodies are usually directed against DNA–histone complexes resulting from cell apoptosis. During the acute EAE phase (7‐20 days after immunization), catalytic antibodies efficiently hydrolysing myelin basic protein (MBP), MOG and DNA are produced with parallel suppression of antibodies hydrolysing histones. We could show that in contrast to MOG, immunization with histone‐DNA results in a reduction of proteinuria, a significant increase in anti‐DNA, anti‐MBP and anti‐MOG antibody titres, as well as an increase in their catalytic activities for antigen hydrolysis, but slightly changes the concentration of cytokines. Contrary to MOG, DNA–histone and DNA‐met‐BSA only stimulated the formation of anti‐DNA antibodies hydrolysing DNA with a long delay (15‐20 days after immunization). Our data indicate that for C57BL/6 mice immunization with DNA‐met‐BSA and DNA–histone complexes may have opposing effects compared to MOG. DNA–histone stimulates the appearance of histone‐hydrolysing abzymes in the acute EAE phase, while abzymes with DNase activity appear at significantly later time‐points. We conclude that MOG, DNA–histone and DNA‐met‐BSA have different effects on numerous bone marrow, cellular, immunological and biochemical parameters of immunized mice, but all antigens finally significantly stimulate the development of the EAE.

## 1 INTRODUCTION

Multiple sclerosis (MS) is the inflammatory and demyelinating disease of the central nervous system (CNS) with perivascular infiltrates composed largely of macrophages and T lymphocytes. Numerous studies support the role of autoimmune mechanisms in the destruction of myelin, while the precise cause of MS remains unknown.^1^ Data indicate that activated myelin‐reactive CD4^+^ T cells may be principal mediators of MS.^1^ Several recent findings also specify an important role of B cells and autoantibodies (auto‐Abs) against myelin autoantigens in MS pathogenesis.^1^, ^2^, ^3^

The appearance of oligoclonal immunoglobulin G (IgG) and increased levels of antibodies (Abs) in the cerebrospinal fluid (CSF), the accumulation of clonal B cells in the CSF, and lesions of MS patients provide key evidence for the involvement of a humoural response in demyelination.^4^ Evidence from recent clinical studies in humans and animal models demonstrates that auto‐Abs against myelin components (involved in antibody‐mediated demyelination^3^) and auto‐Abs against oligodendrocyte progenitor cell protein, which may interfere with remyelination by eliminating or impeding these cells,^5^ seem to play a crucial role in MS immunopathogenesis. In addition to MS‐specific mechanisms, there seem to be some common pathways in the development of different autoimmune diseases (AIDs). For example, the level of lymphocyte apoptosis is usually increased for all AIDs.

In addition to carrying out various intranuclear cell functions, histones act as damage‐molecules in the extracellular space.^6^ Treating animals with exogenous histones leads to systemic inflammation and toxic reactions through activation of Toll‐like receptors and inflammasome pathways. The blood‐level increase in nucleosome fragments and histones is associated with multiple adverse pathophysiological processes including progression in various AIDs and inflammatory diseases.^6^ It has been shown that multifunctional cytokines can influence the human immune system leading to its dysregulation and resulting in the development of different AIDs.^7^ In addition, an important and harmful role in the development of MS^8^ and other AIDs can be attributed to auto‐Abs with catalytic activities (abzymes; ABZs).^9^, ^10^, ^11^, ^12^, ^13^

Artificial abzymes against chemically stable analogues of transition states of different chemical reactions are well described.^9^, ^14^, ^15^, ^16^ In the blood of healthy human beings and animals, it is possible to detect auto‐Abs against different proteins, peptides, DNA, RNA, oligosaccharides and various other components; their titres vary significantly, but these auto‐Abs are usually catalytically inactive.^8^, ^9^, ^10^, ^11^, ^12^, ^13^

Researchers conducted during the last 30 years have revealed different enzymatic activities for auto‐Abs. Auto‐Ab elaboration is a specific feature of AID patients.^8^, ^9^, ^10^, ^11^, ^12^, ^13^ Similar to artificial abzymes, natural ABZs are antibodies against enzyme substrates acting as protein haptens and mimicking transition states of chemical reactions. Anti‐idiotypic ABZs against catalytic enzyme centres also possess catalytic activities.^8^, ^9^, ^10^, ^11^, ^12^, ^13^ Abzymes with many different enzymatic activities were revealed as the earliest and most significant markers of onset and development of various AIDs in humans and mammals^8^, ^9^, ^10^, ^11^, ^12^, ^13^, ^17^, ^18^, ^19^, ^20^, ^21^, ^22^, ^23^, ^24^, ^25^, ^26^, ^27^, ^28^, ^29^, ^30^, ^31^, ^32^ including systemic lupus erythematosus (SLE),^27^, ^28^, ^29^ MS^22^, ^23^, ^24^, ^25^, ^26^, ^30^, ^31^, ^32^ and EAE^20^, ^21^ in experimental mice. Enzymatic activities of ABZs are actually detectable at the pre‐disease stage prior to appearance of obvious AID markers and changes in proteinuria. At that time, antibody titres for various anti‐antigens correspond to a range of indices typical for healthy humans and mice. A detectable level of abzymes can indicate the disease onset or pre‐disease stage, while an increase in catalytic activities follows the development of clear pathological symptoms.^11^, ^12^, ^13^, ^17^, ^18^, ^19^, ^20^, ^21^ To understand the disease, it is therefore essential to identify all possible parallel and complementary mechanisms of MS development.

Current data demonstrate the appearance of myelin basic protein (MBP)‐ and DNA‐hydrolysing abzymes in the blood of patients with MS,^22^, ^23^, ^24^, ^25^, ^26^ SLE^27^, ^28^, ^29^ and several other AIDs.^10^, ^11^, ^12^, ^13^ IgGs from the CSF of MS patients, which hydrolyse MBP, DNA and polysaccharides, are on average 30‐ to 60‐fold higher than those taken from the sera of the same patients.^30^, ^31^, ^32^ In AIDs, abzymes hydrolysing MBP can attack the protein of the myelin‐proteolipid sheath of axons and can play a harmful role in MS, SLE and other disease pathogenesis.^10^, ^11^, ^12^, ^13^ Abzymes with DNase activity are harmful: they are cytotoxic and can penetrate into the nucleus of the cell; they hydrolyse nuclear DNA inducing cell death by apoptosis, which in turn stimulates the development of AIDs.^33^, ^34^, ^35^ Anti‐DNA Abs and ABZs in SLE, MS and other AIDs are usually directed against nucleosomal histone‐DNA complexes, which appear during apoptosis and result in internucleosomal cleavage.^36^

All autoimmune diseases have initially been suggested to originate from defects in hematopoietic stem cells (HSCs).^37^ Later, it was shown that the spontaneous and antigen‐induced development of profound experimental autoimmune encephalomyelitis (EAE)^20^, ^21^ and SLE^17^, ^18^, ^19^ pathologies in autoimmune (AI) prone mice was associated with a specific reorganization of the immune system, including specific changes in the profile of differentiation of bone marrow HSCs in combination with the production of abzymes hydrolysing DNA, ATP, polysaccharides and proteins.

There are several different EAE models, each mimicking a specific facet of MS (for review see^38^). C57BL/6 mice were recently used to study possible mechanisms of spontaneous, myelin oligodendrocyte glycoprotein (MOG)‐ and DNA‐accelerated development of EAE.^20^, ^21^ We confirmed that immunization with MOG and DNA led to an acceleration of EAE development but was associated with various changes in HSC differentiation profiles, lymphocyte proliferation and apoptosis in different organs of mice. The data showed that DNA and MOG may have to some extent antagonistic effects on these parameters and revealed a noticeable delay of EAE development after immunization with DNA. At the same time, as mentioned above, the main immunogen stimulating the development of various AIDs is not DNA but its complex with histones.^6^ Moreover, it is believed that an important role in AID development is played by cytokines.^7^

Similar to SLE pathology, high‐affinity anti‐DNA Abs have recently been identified as a major component of intrathecal IgGs in the brain and CSF cells of MS patients.^39^

Taking all above considerations into account, here we carried out the first extended analysis of the DNA–histone complex effect on the changes of differentiation profiles, lymphocyte proliferation and apoptosis of bone marrow HSCs, relative titres of MBP, DNA, histones and relative activities of abzymes hydrolysing these substrates, as well as on the concentration of cytokines.

## 2 MATERIALS AND METHODS

### 2.1 Reagents

If not stated otherwise reagents, including chemicals, proteins, five histones (equimolar mixture of H4, H3, H2a, H2b and H1), polymeric bovine DNA, the Superdex 200 HR 10/30 column and Protein G‐Sepharose were purchased from Sigma‐Aldrich (Munich, Germany) or GE Healthcare. We used purified human MBP containing 18.5 kD from RCMDT (Moscow, Russia) and MOG_35‐55_ from EZBiolab. These preparations were free from lipids, oligosaccharides, nucleic acids and other possible contaminants. The ELISA test system for the analysis of cytokines was obtained from Biolegend (USA).

### 2.2 Experimental animals

C57BL/6 inbred mice, 3 months of age, were housed under standard pathogen‐free conditions, including a system for protection from viral and bacterial infections, at the Institute of Cytology and Genetics (ICG) mouse breeding facility. All experimental procedures with mice were conducted in accordance with protocols of the Bioethical Committee of the Institute of Cytology and Genetics corresponding to recommendations of the European Committee for the humane principles of work with experimental animals (European Communities Council Directive 86/609/CEE). The Bioethical Committee of Institute of Cytology and Genetics has approved our study in accordance with the European Communities Council Directive 86/609 guidelines.

### 2.3 Immunization of mice

C57BL/6 mice were immunized with a gel (150 μL) combining polymeric DNA complex (94 μg), five histones (92 μg) and Pertussis toxin (0.4 μg). To prepare a conjugate, a solution of 23.6 mg five histones (in 11.8 mL of water) was mixed with 23 mg calf thymus DNA (in 3 mL of water), adding 80 μL of 3 mol/L NaOH (pH 10). After complete dissolution, the mixture was titrated with 1 mol/L hydrochloric acid to pH 7.0 and diluted to 18.8 mL with a physiological solution containing 0.235 mol/L NaCl. As final step a mixture of 18.8 mL of antigen solution, 101.5 μg Pertussis toxin (*Mycobacterium tuberculosis)* in 20* *μL of water, and 18.8 mL complete Freund's adjuvant solution was composed. This mixture was repeatedly passed through a syringe needle to form a homogeneous gel. One hundred microlitres of the gel was injected subcutaneously per mouse; 50 μL into each paw pad. The second immunization of each mouse was performed (after 2 days) in the same manner using a 150‐μL mixture of incomplete Freund's adjuvant containing 0.4 μg of Pertussis toxin. Immunization of mice with MOG and Pertussis toxin, and immunization with DNA without toxin was carried out as described in previously published protocol.^37^ The relative weight of mice and proteinuria (relative concentration of total protein in the urine, mg/mL) was analysed as before.^17^, ^18^, ^19^, ^20^, ^21^ Protein concentration in urine was measured using the Bradford assay with a bovine serum albumin standard. For further experiments including the purification of Abs and analysis of their enzymatic activity, 0.5‐0.8 mL of blood was collected after decapitation using standard approaches.^20^, ^21^

### 2.4 ELISA of anti‐DNA and anti‐protein antibodies

Anti‐MBP and anti‐MOG (for both plasma was diluted 50‐fold), and anti‐DNA (plasma was diluted 100‐fold) antibody concentrations were analysed using ELISA in accordance with previous work.^20^, ^21^ After a consecutive treatment of blood plasma samples with rabbit‐specific anti‐mouse Abs conjugated with horseradish peroxidase, the reaction mixtures were incubated with H_2_O_2_ and tetramethyl benzidine. The reaction was stopped with H_2_SO_4_, and the optical density (A_450_) of the solutions was determined using a Uniskan II plate reader (MTX Lab Systems, USA).

The relative level of auto‐Abs against histones (a mixture of all five histones) was estimated with ELISA test system from EUROIMMUN (Germany) using purified homogeneous IgGs (0.02 mg/mL for one analysis) and adhering to the manufacturer's instructions, as described previously.^40^

The relative concentrations of cytokines (IFNγ, IL‐1β and TNFα) were estimated with ELISA test system from Vector‐best (Novosibirsk, Russia) adhering to the manufacturer's instructions. The plasma samples were diluted 2.5‐fold. Results of the analysis were compared in a calibration curve where the concentrations of cytokines in all samples were expressed as ng/mL.

The relative concentrations of Abs against DNA, MOG, MBP and histones in the samples were expressed as a difference in relative optical density at 450 nm between control and experimental samples. Controls with DNA, MOG, MBP and histones, but without serum samples and with Abs not interacting with the antigens, gave the same results.

### 2.5 IgG purification

Electrophoretically homogeneous mouse IgG preparations were purified using sequential chromatography of plasma proteins on Protein G‐Sepharose and following fast protein liquid chromatography (FPLC) gel filtration as described in.^20^, ^21^, ^22^, ^23^, ^24^, ^25^, ^26^, ^27^, ^28^ To protect Abs from viral and bacterial contamination, they were filtered through syringe‐driven filter units (Millex; 0.1 μm) and kept in sterilized tubes. The absence of viral and bacterial colonies in Ab preparations was checked as shown previously.^31^, ^32^ SDS‐PAGE analysis proving electrophoretic homogeneity of IgGs under non‐reducing conditions (0.1%) was performed in 4‐15% gradient gels. Intact IgGs were visualized using silver staining as previously described.^20^, ^21^, ^22^, ^23^, ^24^, ^25^, ^26^, ^27^, ^28^ To exclude possible artefacts because of canonical enzyme contaminations, IgGs were separated by SDS‐PAGE and their proteolytic and DNase activities were detected using a gel assay according to.^20^, ^21^, ^22^, ^23^, ^24^, ^25^, ^26^, ^27^, ^28^ Activity peaks after electrophoresis were only revealed in the band corresponding to intact IgGs, and there were no other peaks of protein band activities.

### 2.6 DNA‐hydrolysing activity assay

DNase activity of IgGs was analysed according to published methods.^19^, ^21^, ^25^, ^26^ The reaction mixture (20 μL) for analysis of IgG DNase activity contained 20 mmol/L Tris‐HCl (pH 7.5), 20 mmol/L NaCl, 5 mmol/L MgCl_2_, 1 mmol/L ethylenediaminetetraacetic (EDTA), 20 μg/mL supercoiled (sc) pBluescript and 0.03‐0.2 mg/mL of IgGs, and was incubated for 1‐12 hours at 37°C. Products of DNA hydrolysis were analysed by electrophoresis in 0.8% agarose gels. Ethidium bromide‐stained gels were photographed, scanned and analysed using Gel‐Pro Analyzer v9.11. The relative catalytic activity was calculated from the percentage of DNA corresponding to an initial band of intact scDNA and its relaxed form for the experimental conditions, taking into account a distribution of DNA between these bands in the case of the control experiment (incubation of scDNA in the absence of Abs). All initial rates of the reactions were analysed within the linear regions of the time course (15‐40% of DNA hydrolysis) and IgGs concentrations for every preparation (15‐40% of DNA hydrolysis); a complete transition of scDNA to nicked DNA was taken as 100% of the activity. Finally, the relative RAs (% of the hydrolysis) were recalculated to the same standard conditions.

### 2.7 Protease activity assay

The reaction mixtures (10‐40 μL) for analysis of MOG‐, MBP‐ and histone‐hydrolysing activities of IgGs contained 20 mmol/L Tris‐HCl (pH 7.5), 0.7‐1.0 mg/mL of either MOG, MBP or histones and 0.01‐0.2 mg/mL of IgGs. The mixtures were incubated for 1‐21 hours at 37°C. The cleavage products of all proteins were analysed by SDS‐PAGE using 4%‐15% or 12% gradient gels followed by Coomassie R250 staining. The gels were scanned and quantified using GelPro v3.1 software. The RAs of different IgG preparations were estimated as a decrease in the percentage of initial proteins converted to their different hydrolysed forms, taking into account hydrolysis of control proteins incubated without Abs. All measurements (initial rates, % of the hydrolysis) were taken using the conditions of the reaction of the pseudo‐first order within the linear regions of the time course and IgG concentrations (15‐40% hydrolysis of the proteins).

### 2.8 Analysis of bone marrow progenitor cells in culture

Bone marrow samples were flushed out from mouse femurs, and the colony‐forming ability of the bone marrow cells was estimated. Four dishes per mouse (each containing 2 × 10^4^ cells) were cultured in a standard methylcellulose‐based M3434 medium specific for mouse cells (StemCell Technologies, Canada). The medium contained stem cell factor, interleukin (IL)‐3, IL‐6 and erythropoietin (EPO). Relative number of CFU‐GM, CFU‐E, BFU‐E and CFU‐GEMM colonies was calculated after 14 days of sample incubation at 37°C and 5% CO_2_ in a humidified incubator as described previously.^17^, ^18^, ^19^, ^20^, ^21^

### 2.9 Analysis of lymphocyte proliferation

Analysis of lymphocyte proliferation in vitro (sum of B and T cells) was carried out as in.^17^, ^18^, ^19^, ^20^, ^21^ Cells (10^6^/mL) isolated from spleen, bone marrow, lymph nodes and thymus were cultivated in 96‐well flat‐bottom plates (Trasadingen, Switzerland) containing RPMI‐1640 medium supplemented with 10 mM HEPES buffer, 10% of foetal calf serum, 2 mmol/L L‐glutamine, 0.5 mmol/L 2‐mercaptoethanol, 100 μg/mL benzylpenicillin and 80 μg/mL gentamicin. After a 64‐hour incubation period a solution (15 μL) containing 5 mg/mL MTT (tetrazolium dye MTT is 3‐(4,5‐dimethylthiazol‐2‐yl)‐2,5‐diphenyltetrazolium bromide) was added to each well, and plates were incubated at 37°C for an additional 4 hours. Then, plates were centrifuged for 10 minutes at 1200 × *g*, and solutions were removed. Cells were precipitated by the addition of DMSO (200 μL); the mixtures were resuspended and incubated at 23°C for 15 minutes in darkness. The analysis of the relative cell amount was performed spectrophotometrically at 492 nm.

### 2.10 Apoptosis assay

The analysis of cell apoptosis was performed according to.^17^, ^18^, ^19^, ^20^, ^21^ Cells (1 × 10^6^/mL) were incubated in RPMI‐1640 medium containing 10 mmol/L HEPES buffer, foetal calf serum (10%), 2 mmol/L L‐glutamine, 0.5 mmol/L 2‐mercaptoethanol, 100 μg/mL benzylpenicillin and 80 μg/mL gentamicin (5% CO_2_) at 37°C for 48 hours. Then, cell samples were washed using 2 mL phosphate‐buffered saline supplemented with 0.1% NaN_3_ and 0.02% EDTA.^17^, ^18^, ^19^, ^20^, ^21^ The cells were then incubated for 20 minutes at 37°C with a 0.5 mL solution containing 0.1% Triton X‐100, 250 μg/mL RNase and 50 μg/mL propidium iodide (PI). PI‐fluorescence was estimated by flow cytometry (BD FacsVerse). The results are given as the relative percentage of fragmented (hypodiploid) nuclei reflecting the fraction of apoptotic cells.

### 2.11 Statistical analysis

The results are reported as the mean ± SD of at least three to four independent experiments for each mouse, averaged over seven different mice. Differences between the samples and mouse groups were analysed using Student's *t* test; *P *≤* *0.05 was considered to be statistically significant.

## 3 RESULTS

### 3.1 Choosing a model for an extended study of the mechanism of EAE development

MOG‐induced EAE in mice is frequently used as a model of human MS.^38^, ^41^ The immunization of mice with MOG leads to the development of antibodies against MBP, MOG and DNA, and in addition initiates the production of abzymes efficiently hydrolysing not only MBP and MOG but also DNA.^20^, ^21^ When immunizing mice with MOG, the significant acceleration of EAE development strongly depends on the use of Pertussis toxin (PT).^41^ PT was shown to affect the innate immune response; it can cross the blood‐brain‐barrier by increasing its permeability.^42^ Thus, PT can stimulate the development of EAE in mice immunized with MOG because of increasing the blood–brain barrier permeability for MOG.

Previously we have demonstrated that DNA itself is a weak immunogen, but its immunogenic efficacy dramatically increases in different DNA–protein complexes.^43^ Immunization of autoimmune MRL‐lpr/lpr mice with a complex of DNA and methylated BSA, without Pertussis toxin, led to a strong acceleration of the development of mouse SLE, associated with the development of antibodies against DNA and against the abzymes efficiently hydrolysing DNA, ATP and oligosaccharides.^17^, ^18^, ^19^ Comparable to the role of anti‐MBP in SLE, high‐affinity anti‐DNA intrathecal IgGs have been identified as the major components of the cerebrospinal fluid cells and brains of MS patients.^39^ DNase abzymes were revealed in ~80%‐90% of MS patients.^25^, ^26^ Similar to SLE,^34^ DNase abzymes of MS patients^35^ are cytotoxic and induce apoptosis; they therefore can play an important role not only in SLE but also in MS pathogenesis. As SLE and MS patients demonstrate some overlap in medical, biochemical, immunological and psychiatric indexes,^44^ abzymes with DNase activity to some extent play a similar role in the development of both pathologies. Therefore, analogous to the treatment of MRL‐lpr/lpr mice,^17^, ^18^, ^19^ we first immunized C57BL/6 mice with DNA‐met‐BSA without toxin.^21^ As already shown for the treatment with MOG, the immunization of C57BL/6 mice with DNA without Pertussis toxin leads to the development of EAE (similar to the development of SLE in MRL‐lpr/lpr mice), but with a very long delay (approximately 16‐20 days after immunization) until the manifestation of typical disease indicators.^21^ In the absence of toxin, this delay could be because of a reduced blood–brain barrier permeability for the DNA‐met‐BSA complex. Anti‐DNA Abs in AIDs are usually directed against nucleosomal DNA–histone complexes resulting in cell apoptosis.^36^

As these complexes in cooperation with intrinsic human MBP could have an important role in the development of MS, we chose to immunize C57BL/6 mice with a DNA–histone complex combined with Pertussis toxin. In addition to various intranuclear functions, five histones act as damage molecules in the extracellular space.^6^ Taking this into account, we analysed the development of Abs targeting histones and abzymes hydrolysing these proteins.

Finally, our study compares the development of EAE over time using four experimental C57BL/6 mice groups (averaging over seven animals for each time‐point):

- DNA–histone complex + Pertussis toxin treated C57BL/6 mice,
- untreated control C57BL/6 mice,
- MOG + Pertussis toxin immunized C57BL/6 mice.
- DNA‐met‐BSA complex (without Pertussis toxin) immunized C57BL/6 mice

### 3.2 Changes in the weight of mice and proteinuria

**Figure 1:**
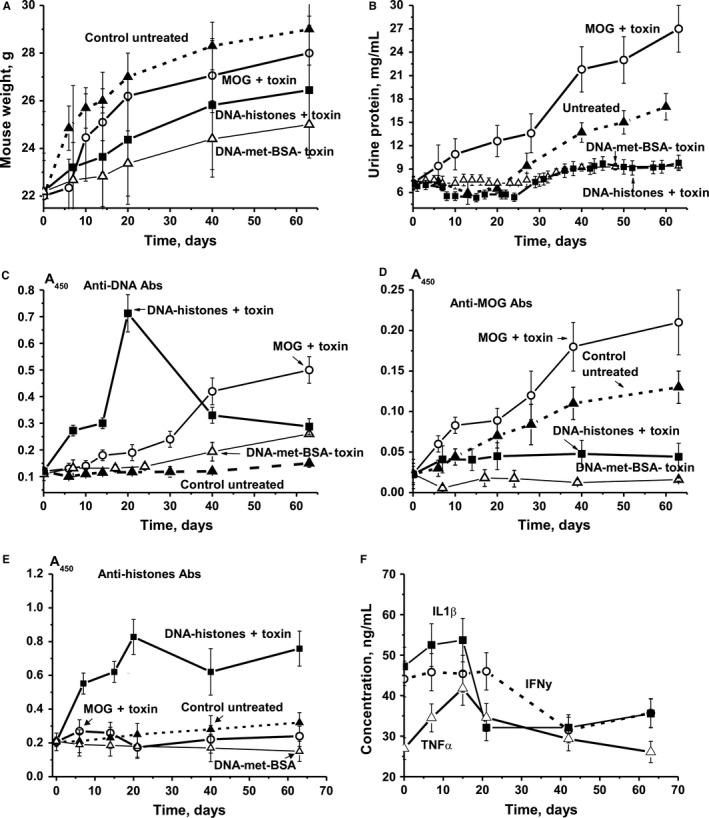
Relative changes in parameters characterizing C57BL/6 EAE mice over time. Changes in body weight (A), proteinuria (B), titres of antibodies to DNA (C), MOG (D), histones (E) and cytokines (F) are shown for groups of untreated control mice, and for those treated with MOG + toxin, DNA‐met‐BSA without toxin, and DNA–histones + toxin. Concentrations of anti‐MOG and anti‐double‐stranded DNA in the sera of C57B2/6 mice were measured using a standard ELISA approach; for details see “Materials and methods”

Changes in the weight of C57BL/6 mice treated with DNA–histones + Pertussis toxin (further referred to as *toxin*) were analysed from the day of immunization (at 3 months of age, time‐point zero) for 63 consecutive days. Control experiments with untreated mice and animals treated with MOG + toxin and with DNA‐met‐BSA were performed in parallel. The data of these experiments are similar to previously published results (comparing the data at 40 days after treatment^20^, ^21^) indicating that the conditions were well reproducible. In contrast to untreated and MOG + toxin‐treated mice, but similar to mice treated with DNA‐met‐BSA complex (decrease in weight compared to control 1.2‐fold; *R *<* *0.05), immunization with DNA–histones + toxin also leads to an essential decrease in animal weight (1.1‐fold; *R *<* *0.05) during the 63‐day observation period (Figure 1A).

In different animal autoimmune models, the development of pathologies usually correlates with an increase in the concentration of proteins in the urine (proteinuria).^17^, ^18^, ^19^, ^20^, ^21^ Control non‐autoimmune CBA and BALB mice do not show proteinuria; at least not during the first 7‐10 months (0.1‐0.12 mg/mL).^17^, ^18^, ^19^, ^20^, ^21^ Healthy MRL‐lpr/lpr mice exhibit low proteinuria (0.38 mg/mL) prior to the development of spontaneous or DNA‐induced pronounced SLE.^17^, ^18^, ^19^, ^20^, ^21^ Initial symptom development in MOG‐induced C57BL/6 mice is usually observed 5‐7 days after their immunization, while the maximum stage of the pathology usually occurs 14‐20 days after the treatment.^41^, ^45^ Interestingly, C57BL/6 mice demonstrate significantly higher proteinuria (up to 8‐12 mg/mL) at three months of age even before immunization with MOG.^46^ While proteinuria increases nearly gradually over time from 7.2 to 27.8 mg/mL in MOG + toxin‐treated mice from day 0 to 63, it almost does not change in untreated control mice until day 20 and then gradually increases to 17.0 mg/mL by day 63. After mice are treated with DNA‐met‐BSA, proteinuria does not markedly change during the first 30 days and then slightly increases to 9.4 mg/mL by day 63. Urine protein levels of mice immunized with DNA–histones + toxin first decrease until day ~25, but then increase and become comparable with the levels seen for the DNA‐met‐BSA complex 30‐63 days after immunization (Figure 1B).

Thus, the treatment of mice with DNA–histone + toxin and DNA‐met‐BSA leads to a statistically significant (*P *<* *0.05) decrease in weight and a decrease in proteinuria compared to untreated and MOG + toxin‐treated EAE mice (Figure 1).

### 3.3 The relative content of anti‐proteins and anti‐DNA Abs

The blood of healthy humans and animals usually contains auto‐Abs against DNA and different proteins in low concentration.^10^, ^11^, ^12^, ^13^ The relative concentrations of Abs against DNA of non‐autoimmune CBA and BALB mice (at 3‐10 months of age) and healthy MRL‐lpr/lpr mice (at 2‐3 months of age) are usually low and vary in the range of 0.03‐0.04 A_450_ units.^33^, ^34^, ^35^ The average concentrations of anti‐DNA Abs at 3 months of age in C57BL/6 mice are ~0.12 A_450_ units, slowly rising approximately 1.3‐fold for untreated mice (0.15 A_450_ units) and 2‐fold for mice immunized with DNA‐met‐BSA (0.26 A_450_ units) until day 63 (Figure 1C). For MOG‐treated mice, the relative concentration of anti‐DNA Abs gradually increases in the course of 63 days and becomes 4.2‐fold higher (*P *<* *0.05) than at the beginning of the experiment (Figure 1C). Surprisingly, immunization of mice with DNA–histones + toxin leads to a significant increase in anti‐DNA Abs at 6‐14 days, and at 20 days (maximum stage of the pathology), it becomes 5.9‐fold higher than at time zero (Figure 1C). In contrast to MOG + toxin‐treated mice, the DNA–histones + toxin‐treated animals reveal a significant decrease in anti‐DNA Ab concentrations at 30‐63 days.

**Figure 2:**
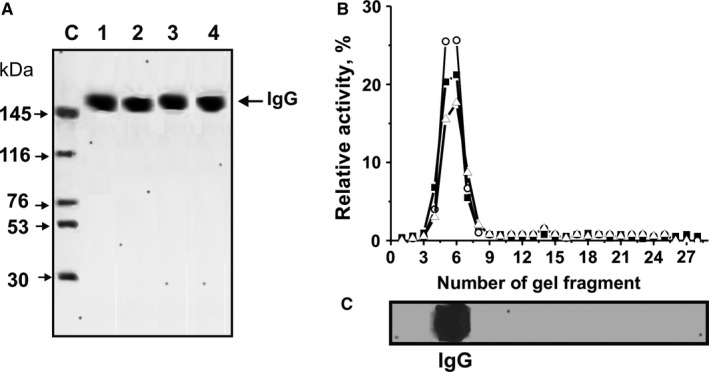
The homogeneity analysis by SDS‐PAGE of 8 μg untret‐IgG_mix_ (lane 1), mog‐IgG_mix_ (lane 2), dna‐IgG_mix_ (lane 3) and hist‐IgG_mix_ (lane 4) under non‐reducing conditions (A, lanes 1‐4); silver staining. The arrow (lane C, A) shows the positions of proteins with known molecular masses. The relative activities (RA, %) of eluate gel fragments in the hydrolysis of DNA (o), MOG (▲and histones (▵) by hist‐IgG_mix_ were estimated using the extracts of gel fragments (2‐3 mm) (B). Panel C shows the position of IgGs. A complete hydrolysis of all substrates after 24 hours of incubation was taken as 100% (B). The errors of the RAs estimations from two independent experiments did not exceed 7%‐10%. For other details, see “Materials and methods”

Figure 1D shows time‐dependent changes in Abs against MOG in the plasma of untreated and treated C57BL/6 mice. The relative average concentration of anti‐MOG Abs of untreated spontaneously diseased mice increases 5.7‐fold (*P *≤* *0.05) in a nearly linear fashion during 63 days. Treatment of mice with MOG + toxin accelerates the increase in anti‐MOG Abs concentration over 6‐10 days, and at day 63 has increased to become 9.1‐fold higher than at time‐point zero (Figure 1D). Immunization with DNA‐met‐BSA without toxin leads to nearly complete suppression of the anti‐MOG antibodies production until day 7 after immunization, and at 63 days they are ~2‐fold lower than at time‐point zero and 17.5‐fold lower compared to the values for MOG + toxin treated mice. In contrast to DNA‐met‐BSA, the treatment with DNA–histones + toxin increases the relative concentration of anti‐MOG antibodies approximately 2‐fold at 7‐20 days and it remains constant until day 63 (Figure 2D). Thus, immunization of mice with DNA–histones + toxin slows down their growth, limits proteinuria and weakly induces the production of anti‐MOG antibodies compared with the high increase for untreated and MOG‐treated mice. It seems that DNA–histones + toxin and complex DNA‐met‐BSA without toxin decelerate the appearance of typical signs characterizing EAE development in the case of spontaneous diseased and MOG + toxin immunized C57BL/6 mice (Figure 1).

Studies have revealed that in addition to various intranuclear functions, five histones (H1, H2a, H2b, H3 and H4) act as damage‐molecules in the extracellular space.^6^ Taking this into account, we have carried out an analysis of changes in concentrations of anti‐histone Abs for the four different mouse groups (Figure 1E). We detected that the concentration of Abs against histones in non‐immunized mice grows very slowly until day 63, increasing 1.5‐fold. Treatment of mice with DNA–histones + toxin leads to a significant 3.8‐fold increase in the concentration of IgGs targeting histones from day 0 to 63. DNA‐met‐BSA and MOG + toxin suppress the formation of anti‐histone IgGs (Figure 1E).

According to the literature,^7^ specific cytokines may play a particular role in the dysregulation of the immune system and the development of AIDs. It is interesting that the immunization of mice with DNA–histones + toxin has relatively weak effects on the concentration of IL1‐β and IFNy showing some increase at days 6‐15. The strongest increase in concentration is found for TNFα. TNFα is a cell signalling cytokine involved in systemic inflammation, and it is one of the cytokines involved in the acute phase reaction.^48^, ^49^ IL1β is also known as lymphocyte activating factor, which is a momentous mediator of the inflammatory response and is involved in a variety of cellular activities, including cell differentiation, proliferation and apoptosis.^49^ Thus, the noticeable increase in the concentrations of TNFα and IL1β in the acute phase of EAE may be influential for the development of autoimmune processes in mice following treatment with DNA–histones + toxin.

### 3.4 The analysis of the criteria directly associated with antibody activities

IgGs were purified from individual mice by affinity chromatography of plasma proteins on Protein G‐Sepharose using special conditions to remove non‐specifically bound proteins as described before.^13^, ^14^, ^15^, ^16^, ^17^, ^18^, ^19^, ^20^, ^21^, ^22^, ^23^, ^24^, ^25^, ^26^, ^27^, ^28^, ^29^ IgGs were then additionally purified using an FPLC gel filtration. For analysis of Ab homogeneity, IgGs from individual mice of each group were used, taking equal amounts of Abs from the plasma of untreated mice (untret‐IgG_mix_), DNA–histones + toxin (hist‐IgG_mix_), MOG + toxin (mog‐IgG_mix_) and DNA‐met‐BSA treated mice (dna‐IgG_mix_). The electrophoretic homogeneity of all typical 150‐kD IgGs is shown by SDS‐PAGE with silver staining (Figure 2A).

Previous studies showed that IgGs from control non‐autoimmune BALB and CBA mice (at 3‐7 months of age) did not possess detectable DNase and protease activities, while IgGs from untreated, MOG + toxin treated and DNA‐met‐BSA treated EAE mice demonstrated detectable or high activity in hydrolysis of DNA, MOG and MBP.^20^, ^21^ In our study, we confirm these data. Within the usual range of data variability, the estimations and average RAs in this study are very well comparable with those obtained previously.^20^, ^21^ After treatment of C57BL/6 mice with DNA–histones + toxin, IgGs are also active in the hydrolysis of all these substrates.

As demonstrated before, the method of isolation of IgGs developed by us excludes contamination with classical enzymes.^17^, ^18^, ^19^, ^20^, ^21^, ^22^, ^23^, ^24^, ^25^, ^26^, ^27^, ^28^, ^29^, ^30^, ^31^, ^32^ Based on several very strict criteria developed previously,^9^, ^10^, ^11^, ^12^, ^13^, ^47^ we have reliably proven that DNase‐, MOG‐, MBP‐hydrolysing activities are intrinsic properties of IgG abzymes from the plasma of spontaneously diseased MOG and DNA‐met‐BSA treated C57BL/6 mice, and they are not because of co‐purified canonical DNases or proteases.^20^, ^21^ To validate our current data set, we additionally prove that IgGs from the plasma of mice treated with DNA–histones + toxin do not contain enzyme impurities. From the hist‐IgG_mix_, the gel strips were cut into 2‐3 mm‐sized fragments after separation from SDS‐PAGE. MOG‐, histones‐ and DNA‐hydrolysing activities were analysed using extracts of proteins from the separated gel fragments (Figure 2D,C). DNA‐, MOG‐, histones‐ and DNA‐hydrolysing activities were found only in the gel fragments containing intact IgGs. SDS destroys any complex proteins, while the electrophoretic mobilities for canonical DNases and proteases (28‐32 kD) are significantly higher than for IgGs (150 kD). Thus, the detection of DNase‐, MOG‐ and histone‐hydrolysing activities in gel fragments corresponds only to intact IgGs (Figure 2B) and provides direct evidence that IgGs of C57BL/6 mice treated with DNA–histones + toxin represent Abs with DNA‐, MOG‐, MBP‐ and histone‐hydrolysing activities.

### 3.5 Time‐dependent changes in antibodies catalytic activities

**Figure 3:**
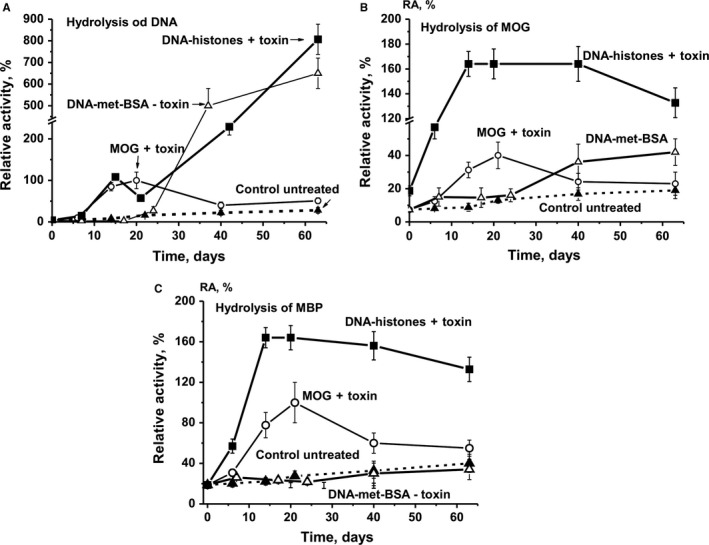
Relative activities of IgGs in the hydrolysis of DNA (A), MOG‐ (B) and MBP (C). The in‐time changes in average values of RAs for IgGs of different mouse groups (each group made up of 7 mice) after their treatment with different antigens are shown on panels A‐C. The error in the rates determined from two experiments conducted for each mouse for all groups did not exceed 7%‐10%

We estimated time‐dependent changes in the average RAs of IgGs corresponding to the four experimental groups, with seven animals each (Figure 3). During the course of 63 days, non‐treated mice demonstrated on average a nearly linear 6.8‐fold increase in DNase activity. After mice were treated with MOG + toxin, we observed a significant 24.4‐fold increase in DNase activity at day 20, corresponding to the maximum stage of the pathology, followed by a significant decrease at 40‐63 days.

Immunization of mice with DNA‐met‐BSA without toxin was associated with remarkably lower DNase activity up to day ~20, increasing only from ~25 to 30 days onwards, and reaching an increase by a factor of 159 until day 63 (Figure 3A). Similar to MOG‐induced effects, treatment of mice with DNA–histones + toxin revealed the first peak of increased DNase activity at 14‐20 days. Comparable to DNA‐met‐BSA, the activity began to grow very rapidly starting at 25‐30 days and at day 63 it was 202‐fold higher than at the onset of the experiment and 8‐fold higher than maximal activity after mice immunization with MOG + toxin (Figure 3A).

Immunization of mice with MOG + toxin led to a statistically significant increase in hydrolysis of MOG (5.4‐fold) and MBP (5.3‐fold) at day 20 (Figure 3B,C). This time period corresponds to the acute stages of EAE disease, which usually manifests itself at 14‐20 days after mice are treated with MOG.^39^, ^40^ At 63 days, the time‐point corresponding to the chronic stage of the disease, MOG‐ and MBP‐hydrolysing activities were significantly decreased (Figure 3A,B).

Similar to untreated control mice, the average activity of IgGs hydrolysing MOG and MBP was only slightly changed up to day 20‐25 after treatment of mice with DNA‐met‐BSA, and for MOG hydrolysis, the activity increased 5.7 times until day 63 (Figure 3B,C). After immunization of mice with DNA–histones + toxin, the RA for the hydrolysis of MBP and MOG increased 8.7‐ and 18‐fold, respectively, at 10‐20 days into the experiment compared to time zero. The RA for the hydrolysis of MBP and MOG was ~1.7‐ and 4‐fold higher, respectively, than the RA after mice treatment with MOG + toxin (Figure 3). Thus, DNA–histones + toxin assimilates the production of abzymes hydrolysing MOG and MBP significantly better than MOG + toxin.

It is expected that immunization of mice with MOG stimulates the production of Abs against MOG as well as the production of abzymes hydrolysing the proteins MOG and MBP. In parallel MOG stimulates, the development of anti‐DNA antibodies and abzymes hydrolysing DNA (Figure 3A). While the immunization of mice with DNA‐met‐BSA resulted in suppressing the development of abzymes against MOG (Figure 3B,C), the treatment with MOG + toxin stimulated the production of DNase abzymes with high activity at 7‐20 days (Figure 3A). Interestingly, after treatment of mice with DNA‐met‐BSA, a strong delay in production of antibodies hydrolysing DNA was observed only after 25‐30 days into the experiment (Figure 3A). In addition, the relative DNase activity of IgGs after treatment of mice with MOG was significantly decreased in the period between days 25 and 63 (Figure 3A). However, during this time, the relative DNase activity of Abs after mice immunization with DNA‐met‐BSA constantly increased, and on day 63 of the experiment, the relative activity was 12.4 times higher than after immunization with MOG and 158 times higher than at the beginning of the experiment. Unlike the immunization of mice with DNA‐met‐BSA, treating them with DNA–histones + toxin resulted in the first peak in IgGs DNase activity at 7‐20 days, comparable to the timing observed for mice immunized with MOG (Figure 3A). In contrast, we observed a nearly linear increase in DNase activity up to day 63, resulting in an 8.2 times higher activity than the maximum activity observed after immunizing the mice with MOG (Figure 3A). Taken together, our data speak in favour of histones and their complex with DNA stimulating immune responses not only against DNA and histones but also against host MBP in rodents.

**Figure 4:**
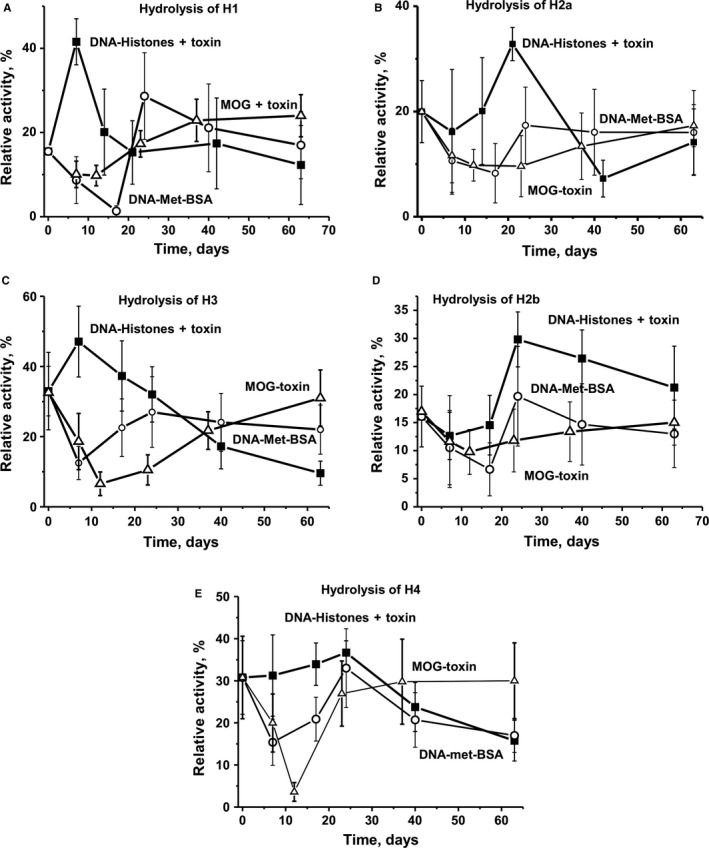
The in‐time changes of RAs protease activities of IgGs corresponding to different mouse groups in the hydrolysis of five various histones are shown on panels A‐E. The error in the rates determined from two experiments conducted for each mouse for all groups did not exceed 7%‐10%

The special role of DNA–histone + toxin in accelerating the production of abzymes after the treatment of mice can be attributed to a fact that complexes of DNA with histones are known as the main immunogens and can powerfully stimulate the formation of anti‐DNA antibodies and abzymes with DNase activity.^36^ In addition, five free histones can stimulate the development of various AIDs on their own.^6^ Figure 1E demonstrates that immunization of mice with DNA–histone + toxin leads to a time‐dependent increase in the relative concentration of Abs against histones. Figure 4 demonstrates time‐dependent changes of RAs for the hydrolysis of five histones (H1, H2a, H2b, H3 and H4) for all four experimental mouse groups. At the beginning of the experiment, the RAs of IgGs for the hydrolysis of five histones decreases in the following order (%): H3 (33) ≥ H4 (31) > H2a (20) > H2b (16.1) ≥ H1 (15.5). For untreated control mice, the changes in the RAs of abzymes for the hydrolysis of the five histones are relatively weak (Figure 4). After immunization with DNA–histone + toxin, RA values increase significantly on day 7; thereafter, the protease activity distinctly decreases for H1 and H3 histones. Interestingly, for the three other histones (H2a, H2b, and H4), the maximum increase in IgGs proteolytic activity occurs at about 15‐25 days, with a decrease in the activity in the following. For mice immunized with MOG + toxin and DNA‐met‐BSA, a sharp decrease in the efficiency of the hydrolysis of all five histones is observed from day 7 to day 20‐25, the activity then increases, and after 63 days it becomes comparable to the activity observed at time zero.

Thus, the production of Abs against DNA, MBP and histones as well as abzymes hydrolysing these antigens slowly but surely occurs even during the spontaneous development of EAE. For mice aged 3 months (time zero), the relative proteolytic activities of the Abs in the hydrolysis of five histones differ at most by a factor of two. Overall, the relative change in IgGs protease activity after the immunization of animals with MOG and DNA‐met‐BSA is comparable but differs for mice treated with a DNA–histone complex.

### 3.6 Hematopoietic progenitor colony formation

In the following, we analysed changes in four types of colonies over time: erythroid burst‐forming unit, early erythroid colonies (BFU‐E); erythroid burst‐forming unit, late erythroid colonies (CFU‐E); granulocytic‐macrophagic colony‐forming unit (CFU‐GM); and granulocytic, erythroid, myeloid colony‐forming unit (CFU‐GEMM).

**Figure 5:**
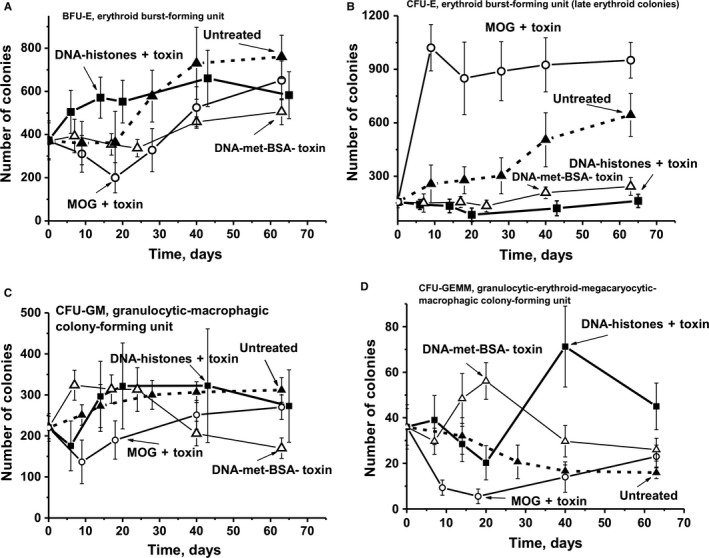
Changes over time in a number of mice brain BFU‐E (A), CFU‐E (B), CFU‐GM (C), and CFU‐GEMM (D) forming colony units are shown for untreated mice, as well as after their treatment with DNA–histone + toxin, a complex of DNA with methylated BSA without toxin (DNA‐met‐BSA), and MOG + toxin. Immunogens used are shown on Panels A‐D

In untreated control mice, the average numbers of BFU‐E and CFU‐E were nearly the same during the first 20‐25 days, and increased 2.1‐ and 4.1‐fold, respectively, by day 63 (Figure 5A,B). Treating mice with MOG + toxin led to changes in BFU‐E and CFU‐E colonies going in opposite directions. The number of BFU‐E colonies decreased 1.9‐fold in the acute phase of EAE (at 6‐20 days), and continuously increased until day 63 by a factor of 1.8 compared to time zero of the experiment (Figure 5A). There was a very sharp 6.5‐fold increase of the relative number of CFU‐E colonies at 6‐10 days, with a slight ~1.1‐fold decrease in their number during days 20‐63 (Figure 5B). In the case of only mice immunized with the DNA–histone + toxin, there was a significant continuous increase in the number of BFU‐E colonies, 1.5‐fold on average from day 7‐63. The mice immunized with DNA‐met‐BSA showed an increase in the number of colonies after 25 days and at day 63 the numbers were approximately 1.4‐fold higher than that at time zero (Figure 5A). Both antigens (DNA–histone + toxin and DNA‐met‐BSA without toxin) resulted in very small changes in the number of CFU‐E colonies during 0‐63 days (Figure 5B).

To some extent, a comparable increase in the number of CFU‐GM colonies was observed in untreated control mice and animals immunized with DNA–histone + toxin and DNA‐met‐BSA without toxin (Figure 5C). Only for mice immunized with MOG + toxin, a sharp 1.6‐fold decrease in the CFU‐GM colonies was observed up to day 10, followed by a slow growth in their number until day 63.

The relative number of CFU‐GEMM colonies gradually decreased in untreated controls and was lower by a factor of 2.3 at day 63 (Figure 5D). While the treatment of mice with MOG + toxin revealed a 6.5‐fold decrease in CFU‐GEMM colonies at day 18‐20 (with a subsequent slight increase in their number until day 63), immunization with DNA‐met‐BSA resulted in a 1.6‐fold increase at day 20 and then decreased 1.4‐fold until day 63 compared to the colonies counted at the beginning of the experiment. Similar to untreated controls and the mice immunized with MOG + toxin, treatment of mice with DNA–histone + toxin induced a 1.8‐fold decrease in the number of CFU‐GEMM colonies, with a sharp 2‐fold increase until day 40, compared to time‐point zero of the experiment.

Thus, untreated control mice reveal some features of a slow spontaneous development of EAE, which is associated with the generation of auto‐Abs against auto‐antigens and abzymes hydrolysing MBP, DNA and histones. Immunization of mice with MOG + toxin, DNA–histone + toxin and DNA‐met‐BSA without toxin leads to an acceleration of the development of EAE and an increased production of harmful antibodies against MBP and DNA as well as elevated concentrations of abzymes hydrolysing MBP, MOG, DNA and histones. In spite the fact that the immunization of C57BL/6 mice with all three antigens finally leads to an acceleration of EAE development, and their characteristics are to some extent comparable regarding immunological and biochemical parameters, the profiles of differentiation of bone marrow stem cells are very specific and different for each immunogen (Figure 5).

### 3.7 Lymphocyte proliferation in different mouse organs

**Figure 6:**
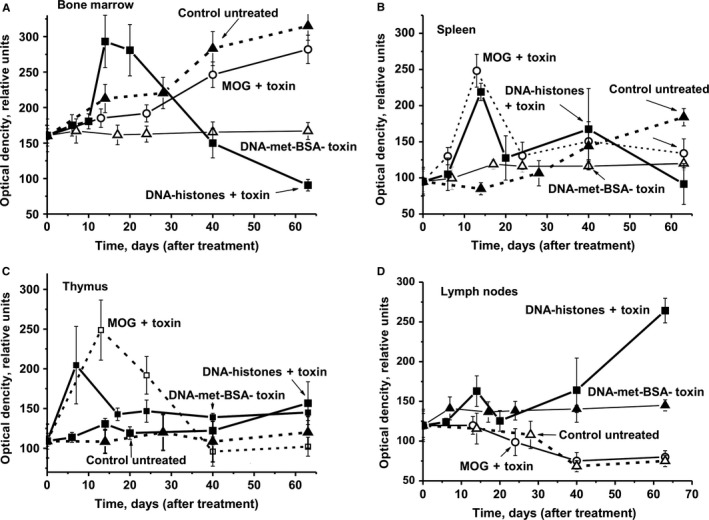
The average over time changes in the optical density reflecting the relative amount of lymphocytes in bone marrow (A), spleen (B), thymus (C) and lymph nodes (D) are shown for untreated mice, as well as after their treatment with DNA–histone + toxin, a complex of DNA with methylated BSA (DNA‐met‐BSA) without toxin and MOG + toxin. Immunogens used are shown on Panels A‐D. The error in the optical density estimation for each mouse for all groups (with seven mice per group) from three independent experiments did not exceed 7%‐10%

It was shown earlier that the production of abzymes is associated not only with a change in profiles of HSCs differentiation but also with an increase in the lymphocyte proliferation level (sum of T and B cells) for MRL‐lpr/lpr and C57BL/6 mice.^17^, ^18^, ^19^, ^20^, ^21^ The relative level of bone marrow lymphocyte proliferation during spontaneous development of EAE slightly differs from proliferation levels after immunization of mice with MOG + toxin, and in both cases almost uniformly increases during the course of 63 days (Figure 6A). After immunizing mice with DNA‐met‐BSA without toxin, the level of proliferation remains almost unchanged during 0‐63 days. This may indicate that during this period DNA‐met‐BSA in the absence of toxin can only minimally penetrate the blood–brain barrier.

The treatment of mice with DNA–histone + toxin resulted in a dramatic increase in the lymphocyte proliferation especially during days 14‐20 (1.7‐1.8‐fold) but then significantly decreased (Figure 6A). This is most likely because of the presence of a toxin which helps DNA–histone + toxin to penetrate the blood–brain barrier and stimulate the synthesis of lymphocyte producing antibodies against MOG, MBP, DNA and histones.

The relative level of spleen lymphocyte proliferation during spontaneous EAE rises slowly and increases 1.9‐fold starting 20 days after immunization until day 63 (Figure 6B). Treating mice with DNA‐met‐BSA without toxin suppresses the proliferation of the spleen lymphocytes, and until day 63 proliferation rates increase only 1.3‐fold. Treating mice with MOG + toxin and DNA–histone + toxin leads to comparable profiles and levels of changes in lymphocyte proliferation in the spleen, with two well‐defined peaks at 14 (2.6‐ and 2.3‐fold, respectively) and 40 days (1.6‐ and 2.3‐fold, respectively).

We cannot exclude that a significant difference in the profiles of spleen lymphocyte proliferation by DNA‐met‐BSA without toxin and DNA–histone + toxin may occur because of the effect of the toxin on the proliferation. Moreover, we cannot rule out that to some extent a stronger proliferation of lymphocytes after immunization with DNA–histone + toxin is associated with the increased immunogenicity of the DNA complex with histones.

### 3.8 Cell apoptosis assay

**Figure 7:**
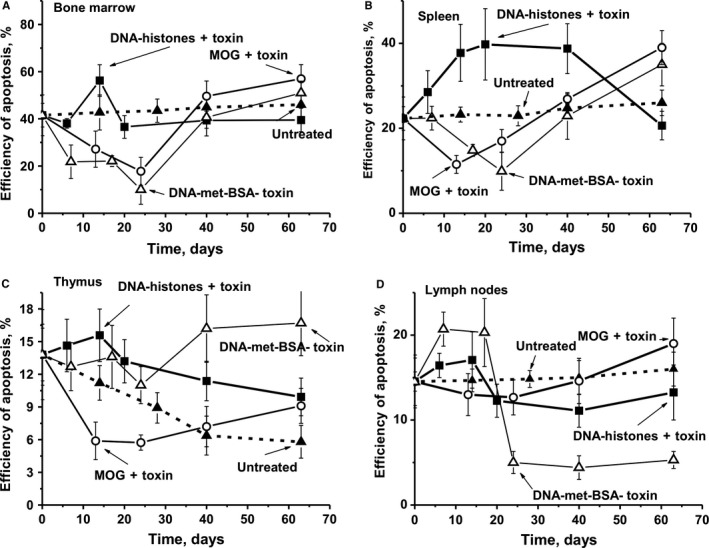
The average changes over time in the relative level of cell apoptosis (%) in bone marrow (A), spleen (B), thymus (C) and lymph nodes (D) for the group of untreated mice, as well as after their treatment with DNA–histone + toxin, DNA‐met‐BSA without toxin, and MOG + toxin. Immunogens used are shown on Panels A‐D. The error in the cell apoptosis estimated for each mouse for all groups from three independent experiments did not exceed 7%‐10%

An important role in the development of AIDs associated with the production of autoantibodies and abzymes may arise from apoptosis of cells synthesizing harmful immunoglobulins.^8^, ^9^, ^10^, ^11^, ^12^, ^13^ During day 0‐63 untreated mice demonstrated a very slow increase in cell apoptosis in all regions except the thymus, in which cell apoptosis is gradually decreased (Figure 7). A comparable decrease in cell apoptosis was observed at 10‐25 days in the bone marrow and the spleen after mice were treated with DNA‐met‐BSA and MOG + toxin (Figure 7A,B). However, after treatment of mice with DNA–histone + toxin, there was the remarkable increase in cell apoptosis in the thymus over time (Figure 7C), compared to a significant decrease in apoptosis in MOG + toxin‐treated mice. Interestingly, MOG + toxin and DNA–histone + toxin revealed opposite effects on apoptosis of thymus cells; the latter antigen stimulating the increase in cell apoptosis in the acute phase of EAE development (Figure 7C). While MOG + toxin only weakly suppressed lymphocyte apoptosis in lymph nodes in the acute disease phase, DNA–histone + toxin and, especially, DNA‐met‐BSA without toxin strongly activated this process, followed by a decrease in apoptosis during days 20‐63.

## 4 DISCUSSION

C57BL/6 mice are used as a model autoimmune‐prone strain that quickly develops EAE symptoms after treatment with MOG.^36^, ^37^ We have earlier shown that the appearance of abzymes exhibiting DNase and proteolytic activities in the blood of humans and animals can be considered a clear indicator of the onset of autoimmune reactions, which are typical for many autoimmune pathologies.^10^, ^11^, ^12^, ^13^, ^14^ Indeed, we determined DNA‐, MBP‐, MOG‐ and histone‐hydrolysing activities of IgGs in three‐month‐old C57BL/6 mice. Previous data^37^ and our current results show that C57BL/6 mice are potentially capable of developing spontaneous autoimmune processes. Treating mice with MOG and DNA–histones leads to a significant increase in all enzymatic activities corresponding to the initial phase (6‐8 days after immunization) and the acute phase (14‐22 days) of EAE. However, after mice are immunized with DNA‐met‐BSA without toxin and DNA–histone + toxin DNase activity strongly increases after a long delay (at 20‐25 days; Figure 3).

The spontaneous development of SLE in MRL‐lpr/lpr and EAE in C57BL/6 mice, as well as their acceleration after treatment with a specific DNA antigen, is characterized by very similar changes in the differentiation profiles of HSCs and is associated with the production of abzymes.^20^, ^21^ It is considered that autoimmune reactions in MS patients, leading to the destruction of myelin, are connected with the formation of auto‐Abs against MBP and other myelin components and play an important role in the development of this disease.^3^, ^5^ However, only high‐affinity anti‐DNA Abs were identified as a major component of intrathecal antibodies in the brain and CSF cells of MS patients.^43^ Therefore, the conclusion was reached that anti‐DNA antibodies are of primary importance for the development of MS.^43^ However, from the literature and our results outlined above, it becomes clear that MS is a multifactorial disease. A genetic pre‐disposition for the disease, as well as autoimmune reactions, catalytic antibodies, relative concentrations of different cytokines and other factors may contribute to the pathogenesis of MS.

In our experiments, mice treated with DNA–histone + toxin exhibit an increase in the concentration of TNFα, which is involved in systemic inflammation and the acute phase reactions,^48^, ^49^ and IL1β, which is an important mediator of the inflammatory response and activator of lymphocytes^49^ in the acute phase of EAE (Figure 1F).

Interestingly, immunization of mice with MOG + toxin leads to the production of Abs and abzymes not only for this protein but also targeting DNA, while the treatment of animals with DNA–histone + toxin induces the development of IgGs hydrolysing histones, DNA, MOG and MBP. It is an important finding that these activities are significantly higher after immunization of mice with antigens used *together* with the toxin (Figure 3), as the latter increases the penetration of antigens through the blood–brain barrier.

As shown earlier^17^, ^18^, ^19^, ^20^, ^21^ and in this work (Figure 5), the production of auto‐antibodies and abzymes with various catalytic activities is associated with a change in the profile of differentiation of bone marrow stem cells. We observe an increase in the level of proliferation of lymphocytes in the bone marrow (Figure 6A). For various autoimmune diseases, usually a high level of apoptosis of harmful lymphocytes is noticed, which increases with the progress of such disease. Figure 7A demonstrates that immunization of mice with MOG + toxin and DNA‐met‐BSA leads to the suppression of apoptosis of bone morrow lymphocytes. Such suppression of lymphocyte apoptosis can lead to an intensive increase of various abnormal cells producing autoantibodies that are harmful for the organisms in the bone marrow and different organs. In favour of this assumption is the fact that the RAs of IgGs for hydrolysis of MBP, DNA and oligosaccharides from the CSF of MS patients are on average 50‐ to 60‐fold higher than Abs from the sera of the same patients.^20^, ^21^, ^22^, ^23^, ^24^, ^25^, ^26^, ^27^, ^28^, ^29^, ^30^, ^31^, ^32^ It seems reasonable to believe that additional amiss differentiation of lymphocytes occurs not only in different organs but already at the level of the bone marrow where the formation of cells producing auto‐Abs and abzymes results in an increase in enzymatic activities of abzymes in comparison with blood sera.

The treatment of mice with DNA–histone + toxin results in a greater increase in the RA of abzymes hydrolysing DNA (Figure 3A), MOG and MBP (Figure 3B,C), as well as histones (Figure 4) compared to that of mice immunized with MOG + toxin. The treatment of mice with DNA–histone + toxin leads to an increase in the efficacy of histone hydrolysis, while powerful suppression of their hydrolysis occurs after the immunization with MOG and DNA‐met‐BSA during the acute phase of EAE development (Figure 4). In the latter cases, the level of antibody activity with histone hydrolysis is much lower than the activity seen for non‐immunized mice (Figure 4). This finding speaks in favour of the competition of histones and mice MBP for interaction with lymphocytes and direction of their following differentiation. As indicated above, anti‐DNA Abs are usually directed against histone‐DNA complexes, resulting in internucleosomal cleavage during apoptosis.^36^ Taking this into account, one would expect that after mice immunization with DNA–histone + toxin, the production of Abs against DNA and histones can occur in parallel approximately at the same time. However, antibodies efficiently hydrolysing histones are detected during the acute phase of EAE (7‐20 days after immunization) (Figure 4), while the initial stage of production of DNase antibodies is observed only after 20‐25 days (Figure 3A). Interestingly, a strong delay (at 18‐20 days) of the initial stage of production of DNA‐hydrolysing antibodies is observed for both antigens containing DNA (DNA‐met‐BSA and histone‐DNA + toxin) (Figure 3A). It should be told that in this situation, that is in the earliest stage of human AIDs, there is usually a production of abzymes against only one specific antigen, for example SLE (anti‐DNA), MS (anti‐MBP) or autoimmune Hashimoto thyroiditis (anti‐thyroglobulin).^8^, ^9^, ^10^, ^11^, ^12^, ^13^ In the later stages of autoimmune pathologies, there is usually a very strong expansion of the abzymes repertoire. Patients may have an extremely large pool of polyclonal Abs hydrolysing many different substrate abzymes containing light chains of kappa‐ and lambda‐types, having different net charges, demonstrating different pH optima, may be metal‐independent or independent on different metal ions, and characterized by different substrate specificities.^8^, ^9^, ^10^, ^11^, ^12^, ^13^ Small fractions of IgGs of four subclasses (IgG1–IgG4) from SLE and MS patients are catalytically active in the hydrolysis of DNA and MBP.^10^, ^11^, ^12^, ^13^, ^27^, ^28^, ^50^, ^51^ This may indicate that various ways of violations of the bone marrow immune system can lead to the production of different autoantibodies and abzymes with diverse enzymatic activities in the case of different AIDs. In principle, SLE and MS are different AIDs. It is believed that humans display differences in genetic predisposition for developing SLE or MS.^52^ However, sometimes identical twins with a hereditary genetic predisposition to MS develop different AIDs, one develops MS and the other SLE.^52^ In addition, SLE and MS patients demonstrate some similarity in the development of medical, biochemical and immunological indexes, including a comparable change in the profiles of brain stem cell differentiation and lymphocyte proliferation.^11^, ^12^, ^13^, ^20^, ^21^, ^53^

The similarity of some immunological indexes between MS and SLE supports the assumption that anti‐MBP with proteolytic activity may occur in SLE patients. Interestingly, DNase Abs are on average ~57‐fold more active in SLE than MS, while MBP‐hydrolysing activity in MS is ~7‐ to 10‐fold higher than in SLE patients.^27^

Taken together, the development of EAE in mice is associated with changes in the profiles of the differentiation of bone marrow stem cells, changes in the levels of lymphocyte proliferation in various organs, significant levels of cell apoptosis, the production of harmful auto‐Abs to DNA, MBP and histones as well as abzymes with various activities and increased concentrations of cytokines.

It is believed that in individual patients with AIDs, the development of initial normal immune reactions can be stimulated by foreign antigens of different bacterial as well as viral infections.^54^, ^55^, ^56^, ^57^ Molecular homology and mimicry between human and viral agents such as Epstein‐Barr, measles, hepatitis B, herpes simplex, influenza and papilloma viruses may be involved in the autoimmune pathogenesis of MS.^58^ Perhaps the antigens of such viruses (and/or some bacteria) are able to penetrate the blood–brain barrier and induce a change in the profile of differentiation and the level of proliferation of bone marrow stem cells in case of long periods of illness and complications. As the abzymes in the bone marrow of MS patients are more active than those in the blood of the same MS patients,^30^, ^31^, ^32^ we can assume that the bone marrow lymphocytes are subjected by viral antigens to a special type of differentiation, leading them to synthesize harmful autoantibodies and abzymes in the bone marrow and in different organs.

Here we show for the first time that rapid development of EAE in mice can be stimulated not only by their immunization with MOG + toxin, but also with DNA‐met‐BSA, and DNA–histone + toxin. We cannot exclude that the initial stages of MS development in humans may be associated with an immunization with a variety of antigens.

## CONFLICT OF INTEREST

The authors declare no conflicts of interest.

## AUTHOR CONTRIBUTIONS

The author(s) have made the following declarations about their contributions: IAO, NAP, VNB and GAN conceived and designed the experiments. KSA, LBT, JAL, AAA and SVS performed the experiments. GAN, TB, SGM, IAO, NAP and SES analysed the data. IAO, NAP, SVA, TB, SGM contributed reagents/materials/analysis tools. GAN, TB, SGM, IAO wrote the manuscript. GAN, TB and SGM developed the theoretical description.
